# The association between undiagnosed diabetes and cognitive function: findings from the China health and retirement longitudinal study

**DOI:** 10.1186/s12902-022-01055-x

**Published:** 2022-06-04

**Authors:** Jiafei Yang, Haiming Xu, Jiangping Li, Yu Zhao, Suzhen Guan, Youjuan Fu, Rui Bao, Zhihong Liu

**Affiliations:** 1 Department of Epidemiology and Health Statistics, School of Public Health and Management, Ningxia Medical University, Yinchuan, 750004 Ningxia Hui Autonomous Region China; 2 Research Center of Health Big Data, Key Laboratory of Environmental Factors and Chronic Disease Control, Yinchuan, 750004 Ningxia Hui Autonomous Region China; 3 Department of Occupational and Environmental Health, School of Public Health and Management, Ningxia Medical University, Yinchuan, 750004 China

**Keywords:** Undiagnosed diabetes, Cognitive function, CHARLS

## Abstract

**Background:**

The cognitive function of people with diabetes has gained an increasing interest in recent years, and this study focuses on exploring the relationship between undiagnosed diabetes and cognitive function among the middle-aged and elderly people in China.

**Methods:**

The data came from the China Health and Retirement Longitudinal Study (CHARLS) which was conducted between July and October 2015. 9855 subjects were enrolled in the study. Executive function and episodic memory were used to assess cognitive function. The subjects were divided into three groups: no diabetes, diagnosed diabetes, and undiagnosed diabetes, and weighted multiple linear regression models were established to evaluate the association of undiagnosed diabetes with cognitive function.

**Results:**

After controlling for covariates, undiagnosed diabetes was statistically associated with executive function (β = −0.215, *P* < 0.01). In the age group of ≥65 years, undiagnosed diabetes was statistically associated with executive function (β = −0.358, *P* < 0.01) and episodic memory (β = −0.356, *P* < 0.01). When adjusting for confounders, no statistically significant associations were found between diagnosed diabetes and cognitive function except in 45-54 age group (β = 0.374, *P* < 0.05).

**Conclusions:**

The cross-sectional study suggested that undiagnosed diabetes was linked to poor cognitive function, especially in the elderly population. Timely diagnosis and active treatment of diabetes are important to reduce the occurrence of cognitive impairment. Further prospective cohort studies are required to articulate the association between undiagnosed diabetes and cognitive function.

## Background

Diabetes mellitus is a chronic and progressive metabolic disorder, having become one of the major public health concerns throughout the world. As the International Diabetes Federation Diabetes Atlas reported in 2019 [1], the global prevalence of diabetes mellitus was 9.3%, about 463 million individuals suffering from diabetes, and estimated to be 10.2% (578 million) by 2030 and 10.9% (700 million) by 2045. However, more than half (50.1%) of people living with hyperglycemia were unaware that they had diabetes in 2019, compared to 49.7% in 201 7[2]. With the accelerating pace of population aging and the increasing prevalence of diabetes mellitus, complications of diabetes mellitus, especially the central nervous system impairment, have attracted more and more attention from the scientific community [3].

Cognitive dysfunction is generally defined as different degrees of cognitive impairment from mild cognitive dysfunction [4], a syndrome defined as cognitive decline greater than expected for an individual’s age and education level but not interfering notably with activities of daily life [5], to dementia which may disallow a patient to function without assistance. A variety of factors have been shown to influence cognitive function [6–8]. The relationship between diabetes and cognition has been reported extensively in many studies [9–11]. However, relatively few studies have been reported paying attention to the effect of undiagnosed diabetes on the relationship between diabetes and cognitive function among the middle-aged and elderly people in China. Furthermore, recent evidence indicated that undiagnosed diabetes could moderate the relationship between diabetes mellitus and cognitive function among Brazilian adults [12]. When undiagnosed diabetes was not correctly classified as either non-diabetes or self-reported diabetes, the relationship was attenuated, or became not statistically significant [13]. Individuals not aware of hyperglycemia have a greater risk of neurocognitive dysfunction than patients having been diagnosed with diabetes [14]. Accordingly, earlier identification and intervention of diabetes mellitus, especially the undiagnosed diabetes, is of great significance to prevent the deterioration of cognitive function [15].

Considering the high prevalence of diabetes and large number of undiagnosed diabetes in China [16], it is of great importance to differentiate the undiagnosed diabetes from the people without diabetes and explore the potential association between undiagnosed diabetes and cognitive function based on a nationally representative health survey and blood test data. In addition, further confirmation of the relationship between different diabetes status and cognitive function was also performed in age-stratified subsamples. The findings may be helpful to alert national health authorities to enhance diabetic screening, promote health education, and improve the cognitive well-being among the middle-aged and elderly population.

## Methods

### Data source and study design

The data utilized in this study came from the 2015 wave of the China Health and Retirement Longitudinal Study (CHARLS), a nationally representative research design of middle-aged and elderly people in China. The project was first conducted in June 2011-March 2012 and attempted to provide a wide range of data from socioeconomic characteristics to health conditions. CHARLS baseline survey was carried out in 150 counties/districts, 450 villages/urban communities using multistage probability sampling. The detailed description of the CHARLS survey design and data collection strategy had been published previously [17, 18]. A second follow-up survey including questionnaires, physical examinations, and blood samples was conducted between July and October 2015. Further information of the blood collection, blood bioassays and descriptive summaries was available elsewhere [19]. The data is available freely at http://charls.pku.edu.cn/.

**Fig. 1:**
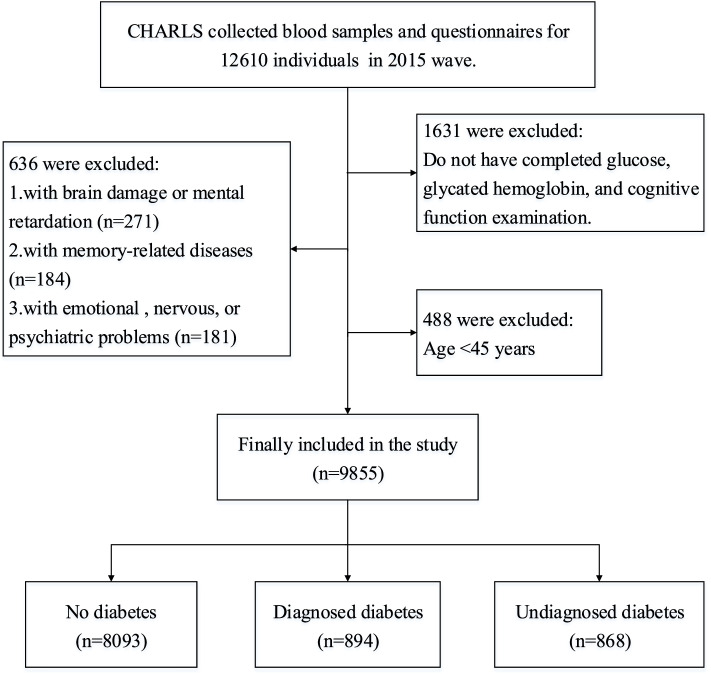
Participants included in the study

Participants included in the study should have complete information of demographic background, cognitive function measurement, blood glucose, glycated hemoglobin, blood lipid examination, blood pressure, BMI, self-rated health status, and depression assessment. Exclusion criteria for this study were: (1) having brain damage or mental retardation, (2) having memory-related diseases such as AD, brain atrophy, Parkinson’s disease, (3) having emotional, nervous, or psychiatric problems. Ultimately, 9855 subjects were included in this study, and they were separated into three groups according to the diabetes status, including 8093 participants without diabetes, 894 participants having been diagnosed with diabetes by a physician or a medical professional, and 868 participants having not yet reported being diagnosed with diabetes but meeting diagnostic criteria. More details about the selection of respondents were presented in Fig. 1.

This study was a secondary analysis of a public database. The original CHARLS was approved by the Ethical Review Committee of Peking University (IRB00001052-11015), and all participants signed the informed consent at the time of participation. The research has been performed in accordance with the Declaration of Helsinki.

### Definition of diabetes status

According to the American Diabetes Association [20], the diagnostic criteria for diabetes mellitus were defined as (1) fasting plasma glucose (FPG) levels ≥126 mg/dL or 2-h plasma glucose (2-h PG) levels in an oral glucose tolerance test ≥200 mg/dL, (2) glycated hemoglobin levels ≥6.5%, (3) classic symptoms of hyperglycemia or hyperglycemic crisis, a random plasma glucose ≥200 mg/dL. The CHARLS survey also had three questions concerning diabetes:“ have you been diagnosed with diabetes or high blood sugar by a doctor?”, “How did you know that you had diabetes, through routine or CHARLS physical examination, or any other?”, and “Are you now taking any of the following treatments to treat diabetes?”. All participants enrolled in the study were classified into three groups: (1) no diabetes; (2) diagnosed diabetes; (3) undiagnosed diabetes. Subjects who had been diagnosed with diabetes by a physician or taking antidiabetic treatments were categorized into “diagnosed diabetes” group. “ Undiagnosed diabetes” group included the individuals fulfilling blood test criteria but without a history of physician diagnosis or taking medication. The remaining subjects were classified into “no diabetes” group.

### Measurement of cognitive function

Based on the design of the American Health Retirement Study (HRS) [21], two core dimensions of cognitive function were assessed in CHARLS, including executive function and episodic memory.

The evaluation of executive function was based on the Telephone Interview of Cognitive Status (TICS-10) and figure redrawing [22]. TICS is a reliable and well-developed screening method of cognitive function [23], which consists of five items on time orientation and five items on the serial-7 s test. The participants were asked to answer today’s date (the month, day, and year), the day of the week, the current season of the year, and then serially subtract 7 from 100 for five times. A score of one was given for each correct answer, while zero for the incorrect. The total TICS scores range from 0 to 10 (Cronbach’s alpha = 0.8) [24]. For the figure redrawing test, interviewees would receive one point if they successfully replicate a picture of two overlapped pentagons shown to them, and zero point if they failed to complete the task. Individual scores were the sum of correct answers, and the measure of executive function theoretically ranges from 0 to 11.

The other cognitive performance was episodic memory evaluated through immediate recall and delayed recall. The immediate word recall test was measured first when ten commonly used words were presented to participants. After completing a 10-item questionnaire on depression, the subjects were again instructed to recall as many of the target words as possible in whatever order. Correctly recalling a word was awarded one point, otherwise, awarded zero. Episodic memory was evaluated by calculating the average score of immediate recall and delayed recall, and the word-recall scores range from 0 to10 [9].

### Covariates

Covariates considered in this study included sociodemographic characteristics, health behaviors, and cognition-related health condition. Sociodemographic characteristics of the participants mainly included gender, age, marital status, the highest level of education, and living areas; health behaviors included smoking habits and alcohol consumption status; the health condition included self-rated health status, BMI level, hypertension, dyslipidemia, and depression.

To examine the effect of age, all participants were divided into three groups: 45-54, 55-64, and ≥ 65 years. Education level was categorized into four groups: illiterate/semi-literate, primary education, middle school education, and high school education or above. Marital status was classified as married or cohabitated, widowed, and other status (including never married, divorced, or separated). Smokers were defined as having smoked more than 100 cigarettes including currently smoking and having quitted, and nonsmokers referred to never smoking. Drinkers were defined as ever drinking alcoholic beverages in the past year, and nondrinkers referred to drinking no alcohol. Based on the Chinese criteria of weight for adults [25], BMI was classified into the following groups: underweight (<18.5 kg/m^2^), normal weight (18.5-23.9 kg/m^2^), overweight (24.0-27.9 kg/m^2^), and obesity (≥28.0 kg/m^2)^ [26].

Blood pressure of the participants was measured three times using the Omron HEM-7200 monitor. According to reference standards established by World Health Organization [27], hypertension was defined as mean systolic blood pressure (SBP) ≥140 mmHg and/or mean diastolic blood pressure (DBP) ≥90 mmHg, taking self-reported physician diagnosis of hypertension into consideration. The Center for Epidemiologic Study Depression Scale (CESD) was adopted as an instrument of depression evaluation in this study [28]. CESD-10 was a short, valid, and reliable scale designed to assess mental disorders for the population in China [29, 30]. 10 questions about the number of negative feelings during the previous week were asked by the interviewers, each term including four grades: 0 (<1 day), 1 (1-2 days), 2 (3-4 days), and 3 (5-7 days). The range of depression scores was 0-30. For present study, a score equal or greater 12 indicated having depression [10, 31]. The lipids including total cholesterol, HDL-cholesterol, LDL-cholesterol, and Triglycerides were measured at Clinical Laboratory of Capital Medical University. Based on the Guidelines for the Prevention and Treatment of Dyslipidemia in China [32], dyslipidemia was defined as TC ≥ 6.216 mmol/L, LDL ≥ 4.138 mmol/L, TG ≥ 2.250 mmol/L, or HD ≤ 1.036 mmol/L. Participants were considered as dyslipidemia when the ratio of total cholesterol to HDL cholesterol ≥5.0 in this study [33]. Individual subjective perception of current health status was also assessed by ranking from 1 (poor), 2 (fair), to 3 (good).

### Statistical analysis

Descriptive statistics were calculated for sociodemographic characteristics, health behaviors, and health conditions. Continuous data were reported as mean ± standard deviation, and categorical data were reported as numbers and proportions. Differences between groups were tested using analysis of variance (ANOVA) or Kruskal-Wallis *H* test for continuous variables and chi-square test for categorical variables. Taking into account the multistage sampling and nonresponse, weighted multiple linear regression models were conducted to determine the potential relationship between undiagnosed diabetes and cognitive function. Baseline models only including covariates were built to screen the significant characteristics. The establishment of crude models was to initially explore the possible relationship between undiagnosed diabetes and cognition. Then, we fit multiple linear regression models controlling for all significant confounding variables to better assess the association of undiagnosed diabetes with cognitive function. In addition, subgroup analyses were carried out to explore heterogeneity. Participants were classified into three groups (45-54,55-64, and ≥ 65 years), and linear regression models were separately constructed to examine whether the relationship was age-dependent. All analyses were conducted in Stata 15.0 (STATA Corporation, College Station, TX, USA), and *P* < 0.05 was regarded as statistically significant.

## Results

### Subjects characteristics

The sociodemographic and health characteristics of the whole sample and the different groups were summarized in Table 1. The mean age of the whole subjects was 60.95 years. More than half of the participants were female (53.41%), more participants (83.11%) were married or living together, and 43.32% of the participants were illiterate or semi-literate. Of the 9855 subjects eventually enrolled in the study, 894 were diagnosed with diabetes, while 868 were not undetected. Compared to nondiabetics, participants with diagnosed or undiagnosed diabetes were more likely to suffer from hypertension (*P* < 0.001), dyslipidemia (*P* < 0.001), and depression (*P* < 0.01). People with undiagnosed diabetes showed poorer cognitive function than those who had been diagnosed with diabetes, with an average score of 5.945 for executive function and an average score of 3.081 for memory function, respectively.

**Table 1:** Sociodemographic and health characteristics of the study subjects

Characteristics	Whole Sample (*n* = 9855)	Diabetes Status			*P*
		No diabetes (*n* = 8093)	Diagnosed DM (*n* = 894)	Undiagnosed DM (*n* = 868)	
Gender (n, %)					0.004
Female	5264 (53.41)	4264 (52.69)	519 (58.05)	481 (55.41)	
Male	4591 (46.59)	3829 (47.31)	375 (41.95)	387 (44.59)	
Age (years, Mean ± SD)	60.95 ± 9.16	60.57 ± 9.17	62.42 ± 8.66	62.96 ± 9.14	<0.001
Age groups (n, %)					<0.001
45-54	2897 (29.40)	2525 (31.20)	189 (21.14)	183 (21.08)	
55-64	3642 (36.95)	2961 (36.59)	354 (39.60)	327 (37.67)	
≥65	3316 (33.65)	2607 (32.21)	351 (39.26)	358 (41.25)	
Marital status (n, %)					<0.001
Married or cohabitated	8190 (83.11)	6744 (83.33)	761 (85.12)	685 (78.92)	
Widowed	1124 (11.40)	890 (11.00)	99 (11.07)	135 (15.55)	
Other status	541 (5.49)	459 (5.67)	34 (3.81)	48 (5.53)	
Educational level (n, %)					0.001
Semi-illiterate	4269 (43.32)	3484 (43.05)	364 (40.72)	421 (48.50)	
Primary school	2299 (23.33)	1904 (23.53)	197 (22.04)	198 (22.81)	
Middle school	2148 (21.80)	1781 (22.00)	202 (22.59)	165 (19.01)	
High school or above	1139 (11.55)	924 (11.42)	131 (14.65)	84 (9.68)	
Smoking status (n, %)					<0.001
Nonsmokers	5851 (59.37)	4779 (59.05)	558 (62.42)	514 (59.22)	
Smokers	4004 (40.63)	3314 (40.95)	336 (37.58)	354 (40.78)	
Alcohol consumption					<0.001
Nondrinkers	6391 (64.85)	5153 (63.67)	651 (72.82)	587 (67.63)	
Drinkers	3464 (35.15)	2940 (36.33)	243 (27.18)	281 (32.37)	
BMI (kg/m^2^, Mean ± SD)	23.90 (3.68)	23.60 (3.55)	25.33 (3.81)	25.20 (4.09)	<0.001
BMI groups (n, %)					<0.001
<18.5	616 (5.59)	550 (6.11)	29 (2.81)	37 (3.77)	
18.5-23.9	5271 (47.86)	4591 (51.02)	345 (33.43)	335 (34.08)	
24.0-27.9	3668 (33.30)	2840 (31.56)	439 (42.54)	389 (39.57)	
≥28.0	1459 (13.25)	1018 (11.31)	219 (21.22)	222 (22.58)	
Depression (n, %)					0.003
No	7450 (75.60)	6157 (76.08)	634 (70.92)	659 (75.92)	
yes	2405 (24.40)	1936 (23.92)	260 (29.08)	209 (24.08)	
Hypertension (n, %)					<0.001
No	7095 (71.99)	5963 (73.68)	578 (64.65)	554 (63.82)	
Yes	2760 (28.01)	2130 (26.32)	316 (35.35)	314 (36.18)	
Dyslipidemia (n, %)					<0.001
No	8245 (83.66)	7016 (86.69)	553 (61.86)	676 (77.88)	
Yes	1610 (16.34)	1077 (13.31)	341 (38.14)	192 (22.12)	
Self-rated health (n, %)					<0.001
Poor	1678 (17.03)	1286 (15.89)	273 (30.54)	119 (13.71)	
Fair	5685 (57.69)	4651 (57.47)	504 (56.37)	530 (61.06)	
Good	2492 (25.28)	2156 (26.64)	117 (13.09)	219 (25.23)	
Living area (n, %)					<0.001
Rural	7344 (74.52)	6140 (75.87)	574 (64.21)	630 (72.58)	
Urban	2511 (25.48)	1953 (24.13)	320 (35.79)	238 (27.42)	
Cognition (Mean ± SD)					
Executive function	6.294 ± 2.469	6.314 ± 2.464	6.457 ± 2.367	5.945 ± 2.587	<0.001
Episodic memory	3.354 ± 1.833	3.382 ± 1.827	3.371 ± 1.747	3.081 ± 1.946	<0.001

### Covariates and cognition

Table 2 showed the relationship between cognitive function and covariates. Being female, the elderly, and living in rural tended to have lower score both in executive function and episodic memory (*P* < 0.001). The participants with hypertension or depression performed worse cognitive function (*P* < 0.001). The people with dyslipidemia performed poor episodic memory (*P* < 0.05), but the difference in executive function was not statistically significant (*P* > 0.05). People having a history of alcohol intake and good self-rated health could get higher scores of episodic memory (*P* < 0.01). The higher the level of education, the better the cognitive function (*P* < 0.001).

**Table 2:** Correlation between covariates and cognition

Characteristics	Executive Function β(t)	Episodic Memory β(t)
Gender (“female” as reference)	0.674^***^(9.38)	−0.267^***^(−3.86)
Age (years)	−0.030^***^(−9.27)	−0.045^***^(−18.45)
Living area (“rural” as reference)	0.711^***^(12.77)	0.412^***^(8.92)
Marital status (“married” as reference)		
Widowed	−0.364^***^(−4.41)	−0.277^***^(−4.72)
Other status	−0.163(−1.56)	−0.088(−0.95)
Educational level	0.843^***^(36.70)	0.558^***^(25.27)
Depression (“no” as reference)	−0.414^***^(−6.65)	−0.309^***^(−6.82)
BMI (kg/m^2^)	0.038^***^(5.35)	0.026^***^(4.72)
Hypertension (“no” as reference)	−0.191^***^(−3.49)	−0.199^***^(−4.54)
Dyslipidemia (“no” as reference)	0.127 (1.77)	0.132^*^(2.50)
Smoking status (“nonsmokers” as reference)	−0.002(−0.04)	−0.017(−0.30)
Alcohol (“nondrinkers” as reference)	0.112 (1.86)	0.211^***^(3.87)
Self-rated health (“fair” as reference)		
Poor	−0.217^**^(−2.85)	−0.006(−0.12)
Good	−0.012(−0.22)	0.131^**^(2.67)

Weighted multiple linear models for executive function and episodic memory in full sample (*n* = 9855)

Statistical significance test: ^***^P < 0.001, ^**^P < 0.01, ^*^P < 0.05

### Diabetes Status and Cognition

We constructed crude models and multiple linear regression models to assess the association between undiagnosed diabetes and cognitive function. The results were presented in Table 3. After adjusting for potential confounding variables discussed in the Table 2, subjects with undiagnosed diabetes showed lower executive function scores compared with nondiabetics(*P* < 0.01). However, this study did not find the statistical association between undiagnosed diabetes and episodic memory based on the full sample (*P* > 0.05).

**Table 3:** Association between different diabetes status and cognition

Variable of interest	Executive Function		Episodic Memory	
	Crude model β(t)	Adjusted model β(t)	Crude model β(t)	Adjusted model β(t)
Diabetes status (“No diabetes” as reference)				
Diagnosed DM	0.171 (1.73)	0.091 (1.05)	−0.025(−0.35)	−0.055(−0.89)
Undiagnosed DM	−0.271^*^(−2.08)	−0.215^**^(−2.60)	−0.186(−1.27)	−0.093(−1.01)

Adjusted model: adjusting significant covariates mentioned in Table 2

Statistical significance test: ^**^P < 0.01, ^*^P < 0.05

### Subgroup analysis

Weighted linear regression models were constructed to analyze the relationship between undiagnosed diabetes and cognition function in age-stratified subsamples. As demonstrated in Table 4, in the age stratum of ≥65 years, after adjusting for potential confounding variables, the subjects with undiagnosed diabetes performed worse cognitive scores both in executive function and episodic memory compared with the nondiabetics (*P* < 0.01). In addition, significant differences in episodic memory performance were found between undiagnosed diabetes and diagnosed diabetes (*P* < 0.05). No such differences were noted in any other age group. However, the estimated coefficient of diagnosed DM in adjusted model was 0.374 (*P* < 0.05) within the 45-54 years of age group.

**Table 4:** Association between diabetes status and cognition among different age groups

Variable of interest	Executive Function		Episodic Memory	
	Crude model β(t)	Adjusted model β(t)	Crude model β(t)	Adjusted model β(t)
Sample in 45-54 years (*n* = 2897)				
Diabetes status (“No diabetes” as reference)				
Diagnosed DM	0.316^*^(2.17)	0.374^*^(2.52)	−0.199(−1.59)	−0.142(−1.19)
Undiagnosed DM	−0.071(−0.40)	0.003 (0.02)	0.067 (0.52)	0.161 (1.28)
Sample in 55-64 years (*n* = 3642)				
Diabetes status (“No diabetes” as reference)				
Diagnosed DM	0.165 (1.03)	0.067 (0.47)	0.037 (0.34)	−0.120(−1.24)
Undiagnosed DM	0.179 (0.83)	−0.163(−1.23)	0.265 (0.94)	0.007 (0.04)
Sample in ≥65 years (*n* = 3316)				
Diabetes status (“No diabetes” as reference)				
Diagnosed DM	0.411^*^(2.41)	−0.090(−0.64)	0.349^**^(2.85)	−0.024(−0.23)
Undiagnosed DM	−0.557^**^(−3.19)	−0.358^**^(−2.67)	−0.443^***^(−3.61)	−0.356^**^(−3.46)

Adjusted model: controlling for significant covariates except age group mentioned in Table 2

Statistical significance test: ^***^P < 0.001, ^**^P < 0.01, ^*^P < 0.05

## Discussion

In the present study, we explored the association between undiagnosed diabetes and cognitive function including executive function and episodic memory based on a nationally representative research of 9855 participants aged 45 years and older in China. Findings showed a high proportion (49.3%) of undiagnosed diabetes among the diabetic population, which was similar to the prevalence reported by the IDF Diabetes Atlas [1] and other studies [34–36]. Mainly, people with undiagnosed diabetes performed worse cognitive function than those who were diagnosed with diabetes and nondiabetics, especially individuals older than 65 years.

Substantial evidence on the relationship between diabetes and cognitive impairment had been reported [37–39]. Diabetes could predict increased incidence of cognitive impairment and dementia [3]. Based on a cohort study, Rawlings et al. [40] found that diabetes in midlife was associated with a 19% greater cognitive decline over 20 years. The pathophysiological mechanisms of cognitive impairment are still obscure, and diabetes is a risk factor for cognitive dysfunction [41]. It is likely that hyperglycemia, insulin resistance, diabetic complications, and brain structural changes play significant roles in the pathogenic process [42–44]. However, this study did not provide adequate evidence to support such hypothesis that people with diabetes had a higher risk of developing cognitive impairment (executive function and episodic memory), even after adjusting for potential confounders that could be related to cognitive function (i.e., age, gender, marriage, education, health status, and other factors). This finding was consistent with previous research [14]. Patients with diabetes were not subjected to cognitive impairment considering that treatment for diabetes might reduce the risk of dementia [45]. Individuals diagnosed with diabetes were treated with medication (such as metformin and ranolazine) or changing behaviors and lifestyle, and all of them could slow down the process of cognitive deterioration [46–49]. Zhang li et al. [9] also reported that untreated diabetes and HbA1c were the potential risk factor for cognitive impairment using the baseline data of CHARLS, and suggested that more longitudinal studies were needed to confirm the effect.

In our study of full sample (*n* = 9855), we found the significant relationship between undiagnosed diabetes and executive function based on multiple linear regression analysis controlling for other confounding factors. When compared to participants without diabetes and diagnosed with diabetes, participants having not yet reported being diagnosed with diabetes showed worse executive function (*P* < 0.01). Undiagnosed diabetes was a potential risk factor for cognitive impairment, especially for executive function. In the same line, previous studies also revealed the effect of undiagnosed diabetes on the association between diabetes and cognitive function. According to the research of Cochar-Soares et al. [12], when undiagnosed diabetics were misidentified as diabetics or nondiabetics, the strength of the relationship between diabetes and impaired memory could been attenuated. Analogously, in a cross-sectional study of 1033 Mexicans aged ≥50 years, Brian Downer et al. [13] reported the similar underestimated association. The association of diabetes with cognitive impairment decreased by 6.3% when undiagnosed diabetics were considered as nondiabetics and by 30.4% when undiagnosed diabetics were categorized as the diabetics. However, interestingly, the association between undiagnosed diabetes and episodic memory was not found in present study (*P* > 0.05). The dissimilarity in the relationship between diabetes and cognitive function may be affected by the differences in sociodemographic characteristics among different regions or countries [50]. Episodic memory was evaluated by immediate word recall and delayed word recall, obviously, which depended largely on the age and the education level of the participants. In the current study, we concluded that age was the factor negatively influencing episodic memory, and the older the age, the poor the episodic memory. Furthermore, people with a higher level of education were more likely to have better memory. These findings suggested that further studies were required to articulate their associations.

Additionally, to examine whether the relationship between undiagnosed diabetes and cognitive function was age-dependent, the participants were classified into three age strata (45-54, 55-64, and ≥ 65 years), and weighted multiple linear regression models were performed separately in the three groups. In the age group of ≥65 years, a similar conclusion was found that people with undiagnosed diabetes showed poorer executive function than those without diabetes. But statistically significant difference in episodic memory performance between undiagnosed diabetes and non-diabetes was also demonstrated. Combining previous results [51–53], our study provided some additional evidence to support the association between undiagnosed diabetes and cognitive function, especially in elderly population. In 45-54 age group and 55-64 age group, participants with undiagnosed diabetes failed to show any such difference in the domain of executive function and episodic memory compared to nondiabetics and diabetics. There had been evidence to support that cognitive impairment was associated both with diabetes duration [54], but also with age [55]. As expected, patients being older or having a longer duration of diabetes mellitus tended to preform worse cognitive function. This suggested that prevention and control of diabetes mellitus in middle age may help to retard cognitive decline later in life [40]. From this point, early screening and diagnosis of this metabolic disturbance could provide an effective way to promote cognitive health of diabetic people. In the three age groups, we did not arrive at the conclusion that people with diabetes performed worse cognitive function than those without diabetes, and this was consistent with the results based on the whole sample. Even in the 45-55 age group, patients diagnosed with diabetes showed better executive function than those without diabetes. Therefore, more in-deep research should be conducted to elaborate the association between diabetes and cognition.

There were some limitations in our study. First, the present results were based on the cross-sectional study design, and causal relationship between undiagnosed diabetes and cognitive function could not be inferred. Therefore, prospective longitudinal studies are needed to confirm the effect of undiagnosed diabetes on cognitive impairment in the near future. Second, although we adjusted for substantial confounding variables, the possibility of residual confounders remained in our analysis. Third, the data could be prone to recall bias and measurement error. Recall bias was intrinsic in the fact that frequent and severe conditions were more likely to be remembered. Measure error was possible in height, weight and blood test. Other limitations including some missing data for variables of interest in CHARLS questionnaire might cause sampling bias. Despite above limitations, the study also has a number of key strengths. First, the findings were based on a nationally representative research design of middle-aged and elderly people in China, which provided a large sample size and good representativeness for our research. Second, strict quality control and standardized measurement were performed in every stage of the research process, so the quality of current study can be guaranteed.

## Conclusions

To conclude, the cross-sectional study suggested that undiagnosed diabetes was the potential risk factor for cognitive dysfunction, especially in the age group of ≥65 years old. In addition, we did not notice that people with diabetes performed worse cognitive function than those without diabetes. Based on current studies, it is an important point that timely diagnosis, early prevention and the optimum treatment of diabetes may contribute to slowing the progress of cognitive deterioration. Thus, governments, communities and public health departments should enhance the diagnosis, treatment, and prevention of diabetes mellitus. Given the large proportion of undiagnosed diabetes and complexity of diabetes complications, more observational, experimental and clinical studies remain to be developed with larger sample sizes, longer follow-up, and more detailed measurements of cognitive function.

## Data Availability

The datasets used during the current study are publicly available at http://charls.pku.edu.cn.

## Abbreviations

CHARLS: China Health and Retirement Longitudinal Study
HRS: American Health Retirement Study
TICS: Telephone Interview of Cognitive Status
CESD: Center for Epidemiologic Study Depression Scale
